# Invasive fungal infections in neutropenic enterocolitis: A systematic analysis of pathogens, incidence, treatment and mortality in adult patients

**DOI:** 10.1186/1471-2334-6-35

**Published:** 2006-02-26

**Authors:** Marcus Gorschlüter, Ulrich Mey, John Strehl, Volker Schmitz, Christian Rabe, Katharina Pauls, Carsten Ziske, Ingo GH Schmidt-Wolf, Axel Glasmacher

**Affiliations:** 1 Department of Internal Medicine I, University of Bonn, Germany; 2 Institute of Pathology, University of Bonn, Germany

## Abstract

**Background:**

Neutropenic enterocolitis is a life-threatening complication most frequently occurring after intensive chemotherapy in acute leukaemias. Gramnegative bacteria constitute the most important group of causative pathogens. Fungi have also been reported, but their practical relevance remains unclear. The guidelines do not address concrete treatment recommendations for fungal neutropenic enterocolitis.

**Methods:**

Here, we conducted a metaanalysis to answer the questions: What are frequency and mortality of fungal neutropenic enterocolitis? Do frequencies and microbiological distribution of causative fungi support empirical antimycotic therapy? Do reported results of antimycotic therapy in documented fungal neutropenic enterocolitis help with the selection of appropriate drugs? Following a systematic search, we extracted and summarised all detail data from the complete literature.

**Results:**

Among 186 articles describing patients with neutropenic enterocolitis, we found 29 reports describing 53 patients with causative fungal pathogens. We found no randomised controlled trial, no good quality cohort study and no good quality case control study on the role of antifungal treatment. The pooled frequency of fungal neutropenic enterocolitis was 6.2% calculated from all 860 reported patients and 3.4% calculated from selected representative studies only. In 94% of the patients, *Candida *spp. were involved. The pooled mortality rate was 81.8%. Most authors did not report or perform antifungal therapy.

**Conclusion:**

In patients with neutropenic enterocolitis, fungal pathogens play a relevant, but secondary role compared to bacteria. Evidence concerning therapy is very poor, but epidemiological data from this study may provide helpful clues to select empiric antifungal therapy in neutropenic enterocolitis.

## Background

Neutropenic enterocolitis is the most important and a highly life-threatening abdominal infection in neutropenic patients. This complication most frequently occurs after intensive chemotherapy in acute leukaemias. The pooled incidence, derived from all available studies, was calculated to be 5.3% in patients hospitalised for haematological malignancies, for high- dose chemotherapy in solid tumors or for aplastic anaemia [1]. The literature provides generally poor evidence on the treatment of neutropenic enterocolitis.

In a previous systematic review, we could not find prospective trials nor case control studies on any part of therapy [1]. The most intensively discussed therapeutic question in the past decades is whether patients with neutropenic enterocolitis should receive conservative or surgical therapy. In contrast, the differential options of medical management consume much lesser space in the discussion sections. Gramnegative bacteria constitute the most important group of causative pathogens [2-5]. There is a consensus that immediate empirical broad spectrum antibiotic therapy is mandatory, but it is unclear which antibiotic is the drug of choice for empirical therapy. After all, application of a modified recommendation of the current IDSA guidelines for the treatment of neutropenic patients with unexplained fever can be regarded as reasonable to select appropriate antibiotics [1,6].

The role of fungi as causative organisms is even more obscure. In conventional reviews on neutropenic enterocolitis, sometimes citations concerning detection of *Candida *spp. in blood cultures can be found [7-9]. In case series and cohort studies, frequencies of causative fungi are either not given or differ largely from 0% up to more than 19% in autopsy studies [10]. In a paediatric study, 16% of the organisms, cultured premortem from blood, were fungi. Moreover, in this report fungal pathogens accounted for 53% of new microorganisms seen at autopsy [11]. Mortality has been reported to be up to 100% in patients with fungaemia [2]. The latter studies suggest a high relevance of fungal pathogens. Changes in the intestinal flora, due to frequent use of broad spectrum antibiotics in neutropenic patients, might support fungal invaders. Surprisingly, the need for an empirical antimycotic therapy is nearly never discussed in reports describing patients with fungal neutropenic enterocolitis. Similarly, guidelines give no clear recommendations for the management of these patients. IDSA guidelines for the use of antimicrobial agents in neutropenic patients with cancer [6], generally, make very few recommendations concerning abdominal infections. Particularly, no statement regarding the indication for antimycotic therapy in neutropenic enterocolitis is given. The use of empirical antimycotic therapy is not recommended as a necessary component of the first-line antimicrobial therapy of neutropenic patient with unexplained fever. Hughes et al. state that, although clinicians disagree as to when, and even if, amphotericin B therapy should be introduced empirically, most believe that the patient who remains febrile and profoundly neutropenic for >5 days, despite the administration of broad-spectrum antibiotics in adequate dosages, is a candidate for antifungal therapy.

On the other hand, there are clinically documented infections in neutropenia in which first line empirical antimycotic therapy can be regarded as standard. The guidelines of the German Society of Haematology and Oncology recommend that in febrile neutropenic patients with severe neutropenia (i.e. lasting for more than 10 days) and lung infiltrates, initial antimicrobial therapy should include amphotericin B [12]. The markedly poorer prognosis of both neutropenic enterocolitis [1] and pneumonia [13] compared to fever of unknown origin (FUO) might suggest that early antifungal could be also beneficial for patients suffering from neutropenic enterocolitis.

For these reasons, we conducted this analysis. Following a systematic search, we extracted and summarised all detail data (if possible, single patient data) from the complete literature to enhance evidence regarding the question whether a routine empirical antimycotic therapy should be administered as a parallel with broad-spectrum antibiotic therapy.

## Methods

The following clinical questions were posed in this systematic review: What is the frequency of invasive fungal infections in neutropenic enterocolitis? What is the mortality of fungal neutropenic enterocolitis? Does the frequency and the microbiological distribution of invasive fungal infections support empirical antimycotic therapy? Do reported results of antimycotic therapy in documented fungal neutropenic enterocolitis help with the selection of appropriate drugs?

### Identification and selection of relevant reports

We performed a computerised search of the MEDLINE database (PubMed version) for appropriate articles, published from 1953 (including Oldmedline) through April 2005, in any language. The keywords used were "neutropenic enterocolitis" (MeSH search term), "neutropenic colitis" and "typhlitis and (neutrop* or granulocyt*)". Subsequently, reference lists of all identified reports, studies and reviews in the field were screened. We excluded articles describing paediatric patients, describing *Clostridium difficile *colitis only, not dealing with neutropenic enterocolitis or duplicate publications. We reviewed the titles and abstracts of all potentially pertinent articles for inclusion. In articles with unclear relevance and in all included articles full text versions were checked. Concerning definition of neutropenic enterocolitis and grading of evidence we refer to our previous study [1]. Studies, describing typhlitis or necrotising enterocolitis during neutropenia, which we considered as sub-entities of neutropenic enterocolitis were included.

### Data extraction

The data were extracted independently by at least two researchers. Discrepancies were solved by consensus discussion including a third researcher. From articles that met inclusion criteria, individual patient data concerning fungal pathogens were extracted. The frequencies of fungal pathogens were analysed and the findings were summarised. In some studies, several patients with fungal neutropenic enterocolitis are described, but the exact number is not determinable. In such circumstances, the minimum number of patients was included in our analysis. These studies were not included in the calculation of the pooled frequency of fungal neutropenic enterocolitis from representative studies (see below).

### Definition of proven fungal infection

Only proven invasive fungal bowel infections were analysed. The definitions adhered to the criteria of Ascioglu et al. [14]. A fungal infection was considered invasive when blood or ascites cultures yielded yeasts with temporally related clinical signs or when fungi could be detected microscopically inside the bowel wall with evidence of associated tissue damage (including ulceration). Detection of fungi in cultures of stool or bowel specimens at autopsy was not considered as proof of invasive fungal infection. Probable or possible fungal infections were not analysed, due to the very heterogeneous reporting of detail data, especially concerning indirect criteria for suspicious patients.

### Further definitions

Laparotomy with no resection was not considered as surgical therapy. The pooled frequency of fungal neutropenic enterocolitis among patients with neutropenic enterocolitis was calculated with two methods: (1) The "pooled frequency of fungal neutropenic enterocolitis from all reported patients" was calculated by division of the sum of all patients with fungal neutropenic enterocolitis from all relevant studies by the sum of all reported patients with neutropenic enterocolitis. (2) The "pooled frequency of fungal neutropenic enterocolitis from representative studies" was calculated by division of the sum of all patients with fungal neutropenic enterocolitis from representative studies by all patients with neutropenic enterocolitis in these studies. Exclusion criteria for these representative studies in this sense were: (a) Studies from the perspective of pathologists (autopsy studies), (b) studies from the perspective of surgeons, (c) studies in which reporting of microbiological data (analysis of pathogens) was insufficient (most radiological studies) and (d) studies describing less than 4 patients with neutropenic enterocolitis.

### Statistics

95%-confidence intervals for proportions (Wilson method) were calculated with the statistical package CIA confidence interval analysis from the book "Statistics with Confidence" 2nd ed. (Eds: Altman, Machin, Bryant and Gardner) [15]. Differences between categorical variables were tested with univariate χ^2^-tests.

## Results

A MEDLINE search, as of 15 March 2005, with the search terms "neutropenic enterocolitis or neutropenic colitis" and "typhlitis and (neutrop* or granulocyt*)" yielded 286 articles. This represented an extension to a previously reported search strategy [1]. After individual review of these titles and abstracts, we excluded 87 articles describing paediatric patients, five articles describing *Clostridium difficile *colitis and 55 other articles, not dealing with neutropenic enterocolitis, reviews without primary data or duplicate publications. After this selection, 139 articles were remaining. Additional hand search in reference lists of these reports and reviews revealed further 47 relevant articles, whose reference lists were also screened. Finally a total of 186 articles were found to be relevant and their full text versions were studied. Among these we found, again, no study on neutropenic enterocolitis that fulfilled the criteria for evidence levels 1a to 3b according to Phillips et al. [16]. We found no systematic review, no randomised controlled trial, no good quality cohort study and no good quality case control study on any part of therapy, particularly, on the role of antifungal treatment. Eighty-four reports of evidence level 4 (case series or poor quality cohort or poor quality case control studies) were detected. One-hundred and two reports of evidence level 5 (expert opinion) were found.

### Studies reporting of fungal neutropenic enterocolitis

In 29 of 186 relevant papers, a total of 53 patients suffering from neutropenic enterocolitis associated with proven invasive fungal infection were reported (Table 1). In 22 of these cases, fungi were detected in blood cultures. In 26 patients, fungi were observed histologically in the bowel wall and in 4 patients, fungi were detected in both types of material. No case of detection of fungi in the ascitic fluid, but one case of isolation of *Candida albicans *in a peritoneal lavage was reported. Underlying diseases of these 53 patients included acute myeloid leukaemia (21 pts.), acute lymphatic leukaemia (3 pts.), acute leukaemia not specified (7 pts.), lymphoma (2 pts.), multiple myeloma (1 pt.), haematological malignancies not specified (8 pts.), malignancies not specified but probably haematological (8 pts.), non-small cell lung cancer (1 pt.) and aplastic anaemia (2 pts.). In 20 of 29 articles, histopathological evidence, which we still consider as the gold standard, confirmed the diagnosis of neutropenic enterocolitis. In 3 articles a clinical diagnosis fulfilling our previously suggested criteria [1] was established. In 6 articles, authors provided several symptom criteria (e.g. abdominal pain), but did not specify whether one criterion or specific combinations was sufficient for diagnosis and whether radiological confirmation was obligatory or not.

**Table 1:** Studies reporting patients with fungal neutropenic enterocolitis

**Study**	**Study type**	**No. pts. with fungal NE**	**Disease**	**OP?**	**Outcome**	**Spec.**	**Pathogen**	**Additional study details**
[45]	CR	1	AML	OP	survived	Histology	*Candida spp. + grampositive cocci*	CR providing sufficient details
[46]	CS2C	1	ALL	OP	survived	Blood culture+ histology	*Candida spp*.	CR providing sufficient details, 1 case of IFI among 2 cases of NE
[47]	PCS	1	HMNS	n.s.	dead	Blood culture	*Candida spp*.	Early detailled autopsy series (69 pts), 1 confirmed fungaemia, 3 questionable cases of IFI
[48]	MCS	4 (or more)	AML	n.s.	n.s.	Blood culture	*Candida spp*.	Acurate retrospective study of D-Xylose malabsorption in110 AML patients. Malabsorption was associated with candidemia but no. of patients with this combination was not completely clear
[22]	CR	1	AML	NOP	survived	Blood culture	*Candida albicans + P.aeruginosa*.	CR providing sufficient details
[19]	MCS	5	AL	n.s.	dead	Histology	*Candida albicans 3 pts Candida glabrata 1 pt Aspergillus fumigatus 1 pt*	Large retrospective study. Important description of bowel wall thickening as negative prognostic factor in 88 pat. with NE. Limitation: Blood culture results are not reported.
[49]	CS3C	1	HMNS	OP	survived	Blood culture	*Fungus n.s*.	CS of 3 pat. with NE (1*fungaemia) among 18 pat. with abdominal complications. Few patient details. Pat. were not consecutive.
[50]	CS2C	1	AML	OP	survived	Blood culture	*Candida spp*.	CR providing sufficient details, 1 case of IFI among 2 cases with NE
[10]	PCS	5 (or more)	MNS	n.s.	dead	Blood culture / histology	*Candida tropicalis 2 pts Candida albicans 1 pt Candida spp. 2 pts*	Interesting autopsy series describing heterogeneity in the pathologic features of NE. Microbiologic data are not clearly linked to individual patients. No. of pat with IFI was not exactly described.
[51]	SCS	1	AA	OP	Dead	Histology	*Candida spp. + bacteria*	Acurate CS of 8 pat. with NE (1*IFI). Pat. were not consecutive.
[52]	CS2C	1	AA	NOP	Dead	Histology	*Candida spp*.	CR providing sufficient details
[53]	CS3C	1	HNHL	OP	survived	Histology	*Candida spp*.	Larger CS of 3 pat. with NE (1*IFI) among 56 pat. with abdominal complications necessitating surgery. Pat. were not consecutive.
[27]	MCS	1	HMNS	NOP	n.s.	Blood culture	*Candida glabrata + Enterococcus spp*.	Cohort study comparing 18 definite and 11 clinically diagnosed consecutive cases of NE. Substantial review.
[4]	MCS	2	AML ALL	NOP	dead	Blood culture 1 /histology 1	*Aspergillus fumigatus 1 pt Candida krusei*	Cohort study describing 13 consecutive cases of NE.
[54]	CS3C	1	AML	NOP	Dead	Histology	*Cryptococcus neoformans*	CR providing sufficient details of an uncommon course and pathogen
[17]	MCS	1	ALL	n.s.	Dead	Blood culture	*Candida spp. (+Aspergillus ?)*	Cohort study describing 13 consecutive cases of NE.
[55]	CS2C	1	MM	OP	Dead	Blood culture	*Candida tropicalis + Staph.aureus + E.coli*	CR providing sufficient details, 1 case of IFI among 2 cases of NE
[21]	CR	1	AML	NOP	Dead	Blood culture	*Candida krusei*	CR providing sufficient details
[20]	MCS	5	AML	OP 1pt n.s.4pts	Dead 1pt n.s.4 pts	Blood culture	*Candida spp. 4 pts Candida guillermondi+ Trichosporon beigelii 1 pt*	Cohort study describing 10 consecutive cases of NE.
[56]	MCS	2	AL	OP1 NOP1	Dead	Histology	*Candida spp*.	Case series describing 10 consecutive cases of NE. Patients were consecutive, but only patients with histologic confirmation were analysed.
[29]	MCS	1	AML	n.s.	survived	Blood culture	*Candida albicans*	Cohort study describing 7 consecutive cases of NE.
[57]	MCS	4	HMNS	n.s.	Dead	Histology	*Candida spp*.	Early large autopsy series. Relatively few individual patient data provided.
[58]	CR	1	NSCLC	OP	Dead	Peritoneal lavage	*Candida albicans*	CR providing sufficient details
[59]	CR	1	AML	OP	survived	Histology	*Fungus n.s. + bacteria n.s*.	Short but sufficiently detailled case report embedded in a review emphasizing nursing actions
[2]	SCS	3	MNS	NOP	Dead	Blood culture	*Candida spp*.	CS including 18 pat. with clinically diagosed NE (3*fungaemia) among 58 pat. with abdominal complications. Few patient details. Pat. were not consecutive.
[60]	PCS	2	AML	NOP	Dead	Histology	*Candida spp*.	Early smaller (6 patients with NE) but detailled autopsy case series.
[61]	RCS	2	AML	OP	Dead	Histology + Blood culture 1pt Histology 1pt	*Candida spp. 1pt Candida spp.+ bacteria n.s. 1 pt*	Smaller (6 patients with NE) but detailled radiological case series.
[62]	MCS	1 or more	HMNS	NOP	Dead	Histology	*Fungus n.s*	Cohort study describing 34 consecutive cases of NE. No. of pat with IFI was not exactly described.
[28]	SCS	1	LY	NOP	Dead	Histology	*Candida spp*.	Case series describing 22 patients with NE. Pat. were not consecutive. Relatively few individual microbiological data provided.Very good review.
Total 29		53						

ns, not specified; NE, neutropenic enterocolitis; IFI, invasive fungal infection; AML, acute myeloid leukaemia; ALL, acute lymphatic leukemia; MM, multiple myeloma;

AL, acute leukaemia; CL, chronic leukemias; MNS, malignancy not specified; HMNS, haematological malignancy not specified; LY, lymphoma; NCCLC, non-small cell lung cancer; pts patients; OP, operated; NOP, not operated; CR, case report; CS3C, case series with 3 cases; MCS, medical case series; PCS, pathological case series; RCS radiological case series; SCS, surgical case series

### Causative pathogens

In 3 patients, invasive fungal infection with no further specification of the pathogen was diagnosed. In 94% (47/50) of the remaining patients, *Candida *spp. were involved. In 32 patients, the pathogens were reported as *Candida *spp. without a species description. In one of these patients, the blood culture contained also *Aspergillus *spp. [17]. However, a single detection of *Aspergillus *spp. in blood cultures is commonly not considered as proof of an invasive fungal infection due to the risk of false-positive results by environmental contamination [14,18]. In 18 patients, a species description of the fungal pathogen was provided: *Candida albicans *(7 pat.), *Aspergillus fumigatus *(2 pat.), *Candida tropicalis *(3 pat.), *Candida glabrata *(2 pat.), *Candida krusei *(2 pt.), *Cryptococcus neoformans *(1 pt.). *Candida guillermondi *+ *Trichosporon beigelii *in the same blood culture (1 pt.). In 10 patients, it was not clearly reported whether also bacteria were involved in the infection. In 30.2% (13/43) of the remaining patients, equally causative bacteria were observed either in blood cultures or in the bowel wall (species not specified (8 pts.), gram-positive cocci (1 pt.), *Enterococcus *sp. (1 pt.), *Pseudomonas aeruginosa *(1 pt.), *Staph. aureus *+ *Escherichia coli *(1 pt.), *P. aeruginosa + E. coli + Klebsiella pneumoniae *(1 pt.)). In 30 patients, fungi were considered to be exclusively causative.

One of the patients of our institution had a proven invasive small bowel infection with *Aspergillus fumigatus *[4]. Neutropenic enterocolitis caused by moulds is extremely rare. Only one further case is published [19]. Our patient was a 63-year old man with AML already neutropenic on admission (WBC count: 0.1 G/l without recovery). On day 9, after chemotherapy with fludarabine, high-dose cytarabine and dexamethasone (FLAG-Ida) he developed paralytic subileus, abdominal pain, vomiting, meteorism and fever. There was no improvement with broad-spectrum antibiotics and amphotericin B for 11 days. Death occurred on day 25 after chemotherapy due to sepsis. Autopsy revealed an ulcer in the middle of the jejunum, with a diameter of 3 cm, caused by *Aspergillus fumigatus *and the same pathogen as causative agent of concomitant pneumonia [4].

### Therapy and prognosis

In 25 patients, it was unclear whether they were treated conservatively or surgically. Of the remaining 28 patients, 13 patients (46.4%) were reported to be treated surgically and 15 (53.6%) were managed conservatively. In 33 patients, it was not described that any antifungal therapy was given. In 20 patients, with proven fungal neutropenic enterocolitis at least some information regarding antifungal therapy was provided: The antimycotic therapy of 4 surviving patients is described below. Regarding the fatal cases, Cartoni et al. reported that empirical antibiotic therapy was given followed by empirical antifungal agents in selected cases. We assume that this was the case in five patients, which we included as fungal neutropenic enterocolitis. In the study of Micozzi et al., selected patients, probably also those five patients which we considered to have fungal neutropenic enterocolitis, received amphotericin B [20]. It was not specified whether therapy was empirical or following microbiological documentation. Starnes et al. reported that antimycotics were not successful in all 3 patients with candidaemia [2]. In our own series, in both patients, treatment with amphotericin B (1 mg/kg/d) was not successful [4]. McIlroy et al. observed a break-through infection with *Candida krusei *despite empirical fluconazole in one patient with clinical diagosis of neutropenic enterocolitis. Unfortunately, even a change to amphotericin B was not successful [21].

In 9 of the 53 patients, it was unclear whether they survived or died in the course of infection. From the remaining 44 patients, 36 died and the pooled mortality rate was calculated to be 81.8% 36/44 pts., 95%-CI: 68–91 %). Seven of them had been operated on. Thirteen patients were treated conservatively. In 16 patients the type of treatment was not specified. Eight patients (18.2%) survived. Six of them had been operated on. One patient was treated conservatively, in one patient the type of treatment was not specified. In 4 of the 8 survivors, antifungal therapy was not described. Three patients received antifungal therapy, which was not specified. One patient received fluconazole empirically, which was changed in the course to amphotericin B [22]. Three patients were described to recover early from neutropenia, in the other 5, no data concerning recovery of haematopoesis were provided.

### Calculation of pooled frequency rates

The pooled frequency of fungal neutropenic enterocolitis among patients with neutropenic enterocolitis was calculated with two methods. Both have advantages and disadvantages:

1. The pooled frequency of fungal neutropenic enterocolitis from all reported patients was calculated to be 6.2% (53/860 95%-CI: 4.7–8.0%). This method includes all available information, but is limited by multiple selection biases (selection of more severe autopsied or operated cases).

2. The pooled frequency of fungal neutropenic enterocolitis from 12 representative studies (Table 2) was calculated to be 3.4% (5/146; 95%-CI: 1.5–7.8%). This method includes only a smaller part of available information, but selection bias is reduced as much as possible. However, fungal neutropenic enterocolitis may be underestimated, since only few histologic specimens were included and the sensitivity of the diagnostic methods is limited.

**Table 2:** Representative studies included for calculation of pooled frequency of fungal neutropenic enterocolitis

**Study**	**Study type**	**Underlying diseases**	**No. FNE**	**No. NE**	**Additional study details**
[5]	Retrospective MCS	AL and CL	0	20	Detailled cohort study describing 20 consecutive patients with NE
[27]	Retospective MCS	Mostly AL (some solid tumors).	1	29	Cohort study comparing 18 definite and 11 clinically diagnosed consecutive cases of NE. Substantial review.
[4]	Retrospective MCS	AL. Some other abdominal infections in other neutropenic pts. are described	2	13	Cohort study describing 13 consecutive cases of NE.
[23]	Prospective diagnostic study	AL	0	4	Relatively small but prospective ultrasound -based diagnostic study applying exactly defined diagnostic criteria
[63]	Retrospective MCS	AML	0	10	Cohort study describing 10 consecutive cases of NE. Relatively brief, but sufficient data
[64]	Retrospective MCS	AL	0	9	Detailled cohort study describing 9 consecutive patients with NE
[65]	Retrospective MCS	Metastatic breast cancer receiving docetaxel-based chemotherapy.	0	4	Small cohort study in taxane-exposed patients. Relatively brief, but sufficient data.
[17]	Retrospective MCS	AL	1	13	Cohort study describing 13 consecutive cases of NE.
[66]	Retrospective MCS	Cancer pts receiving taxane-based chemotherapy	0	5	Cohort study describing few consecutive patients with NE among 4600 courses of taxane-based therapy. Relatively brief, but sufficient data.
[18]	Retrospective MCS	AML	0	12	Detailled cohort study describing 12 consecutive patients with NE within an analysis of all infections in pat. receiving cheomtherapy
[29]	Retrospective MCS	AML	1	7	Cohort study describing 7 consecutive cases of NE.
[3]	Retrospective SCS reporting of consecutive patients	AL and some pts with CL.	0	20	The only surgical cohort study describing consecutive patients. Relatively brief, but sufficient data.
Total			5	146	

FNE, fungal neutropenic enterocolitis; ns, not specified; NE, neutropenic enterocolitis; AML, acute myeloid leukaemia; AL, acute leukaemia; CL chronic leukemias; pts, patient; MCS, medical case series; SCS, surgical case series

### Prospective diagnostic surveillance studies

Four of 186 papers were prospective diagnostic surveillance studies [23-26]. In none of the 28 patients of these studies, was a proven invasive fungal enterocolitis described. In one study, Girmenia et al. assessed the role of *Candida spp*. in 20 consecutive patients with neutropenic enterocolitis by an assay for *Candida *mannoprotein antigen [24].

## Discussion

This is the first analysis on the relevance of fungal pathogens in neutropenic enterocolitis of adults, based on a systematic analysis of the literature (including our own primary data). The approach was to extract the complete detail data from the studies and to increase evidence by pooling analyses. The maximum number of clearly described patients in a single study was 5, which is to low to perform conclusive statistics concerning frequency, mortality, spectrum of causative species or therapy. A discussion about this question is lacking in the literature, especially in contrast to the extensive debate concerning the role of surgery.

Our study revealed several important results: (1) We found that a total number of 53 patients with fungal neutropenic enterocolitis in adults was described in 29 papers. (2) The pooled frequency of fungal neutropenic enterocolitis was 6.2% calculated from all reported 860 patients and 3.4% calculated from representative studies only. (3) In 94% of the patients *Candida *spp. were involved. (4) The pooled mortality rate was 81.8% (5) In the majority of the patients with fungal neutropenic enterocolitis, no antimycotic therapy was described and possibly not administered.

The frequencies of invasive fungal infections in neutropenic enterocolitis were lower than expected. In our own patients from two studies, we observed fungal neutropenic enterocolitis in 11.8% (2/17) [4,23]. Autopsy studies reported frequencies up to more than 19% [10]. However, several selection and detection biases of these methods of frequency calculation have to be considered. On one hand, fungal neutropenic enterocolitis is probably underestimated, since histology, as the most specific diagnostic method, is rarely obtained in living patients. On the other hand, patients with more severe courses may be selected because of the inclusion of surgical or pathological studies that did not describe consecutive patients. Furthermore, the diagnostic criteria in studies concerning neutropenic enterocolitis are generally heterogeneous. However, in most reported cases of invasive fungal infection, neutropenic enterocolitis was confirmed histopathologically (Table 1).

Fungal infections other than *Candida spp*., particularly *Aspergillus fumigatus*, are rare exceptions. Unfortunately, only in a minority of reported cases, a species confirmation is available. Among these patients, *Candida albicans *occurred most frequently, but in some patients, fluconazole-resistant species such as *Candida glabrata *and *Candida krusei *have been observed. In approximately half of the patients, diagnosis of invasive fungal infection is established by blood culture and in the other half by histology. Bacteria are clearly the most important group of pathogens in neutropenic enterocolitis. Rates of bacteraemia between 34% [27] and 82% [28] have been reported in larger series. In most patients, development of neutropenic enterocolitis is probably a multifactorial process. In our analysis, the term "causative pathogen" does not mean "exclusively causative". We recognize that mostly secondary infection of injured mucosa is responsible for deterioration, complications and poorer outcome of the patients. Furthermore, from a clinical and practical point of view, the treatable factor infection and not hardly treatable factors such as toxic damage should be stressed.

The mortality of fungal neutropenic enterocolitis was very high (81.8%). This pooled mortality rate may be biased and slightly overestimated due to inclusion of 8 patients from autopsy case series. Less severe cases may be moderately underrepresented. However, it must be emphasised that the course of fungal neutropenic enterocolitis is not invariably fatal. Eight patients survived and the description of a 100% mortality rate reported by Starnes et al. and cited by others [2,29] can not be confirmed. It was striking, that in the majority of cases with fungal neutropenic enterocolitis, authors did not report or discuss the use of antimycotics. One might speculate that this obviously low grade of consideration of antifungal therapy in neutropenic enterocolitis might have contributed to the high mortality rate.

Infections from *Aspergillus *spp. are very rare exceptions (despite observation of two patients including one with jejunal ulceration by *Aspergillus *in one of our studies [4]). This rarity may be explained by the fact that *Aspergillus *infections are typically acquired by inhalation and invasive growth in the respiratory tract.

Evidence concerning empirical antifungal therapy of neutropenic enterocolitis is still very limited. We found that neither prospective nor high quality retrospective studies concerning this issue in neutropenic enterocolitis are available. In 33 patients with fungal neutropenic enterocolitis, in reports of evidence levels 4 and 5, it was not described that any antifungal therapy was given. In 20 patients with proven fungal neutropenic enterocolitis more or less precise information regarding empirical or microbiologically induced antifungal therapy was provided. Only in two of these patients empirical antimycotic therapy (fluconazole) was clearly reported [21,22]. Many other patients probably received antimycotics only after detection of fungaemia. Unfortunately, patient numbers and data quality are not sufficient to analyse, statistically, the success of antimycotic therapies, especially empirical use. Our analysis is limited by lack of additional individual information from the authors on antifungal therapy.

Possibly, many authors see no need for empirical antifungal therapy. Only few authors of the relevant studies recommended empirical antifungal therapy [20,24,30]. Girmenia et al. suggested a semi-empirical selective therapy with fluconazole based on the detection of *Candida *mannoproteinaemia [24]. Micozzi et al. pointed out that empirical administration of fluconazole in all febrile patients could represent a way to prevent neutropenic enterocolitis [20]. Wach recommended that conservative therapy of neutropenic enterocolitis should include antimycotic drugs but did not provide more specific recommendations [30]. D'Amato et al. stated that antifungal treatment should be considered [31].

In the NCCN practice guidelines for fever and neutropenia "consideration of antifungal coverage" is recommended for febrile neutropenic patients with abdominal pain or diarrhea [32]. In the IDSA guidelines for the use of antimicrobial agents in neutropenic patients with cancer, no specific recommendation concerning fungal neutropenic enterocolitis is provided [6]. However, one might have the opinion that these patients fall within the scope of the sections of these IDSA guidelines referring to the treatment of neutropenic patients with unexplained fever. They state that, although clinicians disagree as to when, and even if, amphotericin B therapy should be introduced empirically, most believe that the patient who remains febrile and profoundly neutropenic for >5 days despite the administration of broad-spectrum antibiotics in adequate dosages, is a candidate for antifungal therapy [6].

So, should empirical antimycotic therapy in neutropenic enterocolitis be recommended? If the answer is yes, when should antimycotic therapy be started? Which drugs should be used? We believe that application of the above recommendations for FUO to our subgroup of patients is justified on principle. All patients who remain febrile and profoundly neutropenic for >5, days including those with neutropenic enterocolitis should receive amphotericin B empirically. Some patients, who are already febrile several days before clinical signs of neutropenic enterocolitis develop, will receive amphotericin B earlier as 6 days after diagnosis of neutropenic enterocolitis.

The question whether the frequency and the microbiological distribution of invasive fungal infections support immediate empirical antimycotic therapy can not be answered clearly from the literature data. If the moderately low frequency of fungal pathogens is taken into consideration, clinicians might not be willing to start empirical antimycotic therapy with amphotericin B only based on the diagnosis of neutropenic enterocolitis, immediately on the first day of fever. Therefore, an earlier start of empirical antimycotic therapy (like in patients with pulmonary infiltrates) would not be preferred by many.

However, some arguments could be made in favour of an empirical use of fluconazole in neutropenic patients with enterocolitis: (1) The drug has a low toxicity. (2) *Candida albicans *is the leading fungal pathogen. (3) Patients with neutropenic enterocolitis and a positive *Candida *mannoprotein test treated with fluconazole have been shown to have a good prognosis [24]. (4) Fluconazole is an effective drug in clinically stable, non-neutropenic patients with candidaemia [33].

However, fluconazole is not a safe choice for treating non-albicans candidaemia, especially when *Candida krusei *(primary resistance) or *Candida glabrata *(dose-dependent sensitivity) have been identified [34]. Many epidemiological studies indicate that the proportion of non-albicans *Candida *species is clearly increasing and around 50–60 % [35-37]. Also, the empirical use of fluconazole must be restricted to patients who did not receive azoles as antifungal prophylaxis, since no improvement of antimycotic coverage by fluconazole can be expected. Currently, a major part of patients will be pretreated with itraconazole. Our own meta-analysis suggests such an antimycotic prophylaxis in long term neutropenic patients [38], although this practice is still a matter of debate [39] and although its impact on the incidence of neutropenic enterocolitis is unclear. Furthermore – and this a very strong argument against fluconazole for neutropenic enterocolitis – many patients suffer from multiple infections or are at least at high risk to develop a mould infection which would not be covered by fluconazole. All substances that have demonstrated efficacy in valid clinical trials of empirical antifungal therapy for FUO are active against *Aspergillus *spp. like liposomal amphotericin B [40], itraconazole [41] and caspofungin [42].

Unfortunately, no data are available on the use of itraconazole or other novel antimycotics, such as voriconazole and caspofungin, in patients with proven fungal neutropenic enterocolitis nor on the empirical use of these agents in this entity. A prospective multicenter study comparing empirical therapies, antibacterial agent vs. the same antibacterial agent plus antimycotic, is warranted.

In our opinion, in patients with evidence of invasive fungal neutropenic enterocolitis (in most cases by detection of candidaemia) administration of amphotericin B, caspofungin or voriconazole are appropriate choices. However, in contrast to amphotericin B [4,22], we found no paper reporting experiences with caspofungin or voriconazole in fungal neutropenic enterocolitis, but only encouraging results from candidaemia trials [43,44]. Of these latter drugs, caspofungin is indicated for patients with renal dysfunction.

## Conclusion

In patients with neutropenic enterocolitis, fungal pathogens play a relevant, but secondary role compared to bacteria. The frequency of invasive fungal neutropenic enterocolitis is probably around 5% and its mortality around 70–80%. The frequency may be underestimated, since only few histological specimens are obtained in living patients and the sensitivity of the diagnostic methods is limited. Early empirical antimycotic therapy may be therefore considered in neutropenic enterocolitis. However, evidence concerning therapy is very poor, since most authors did not report or apply antifungal therapy. A clear recommendation for empirical antimycotic therapy outside the context of prolonged fever can not be made.

## Competing interests

AG has received research grants from Ortho-Biotech and MSD Sharp & Dohme as well as speakers honoraria from MSD Sharp & Dohme, Ortho-Biotech and Pfizer and served on advisory boards of MSD Sharp & Dohme, Ortho-Biotech. UM has received speakers honoraria from Ortho-Biotech. The other authors declare that they have no competing interests'.

## Authors' contributions

MG was responsible for the design and the statistical analysis of the project; MG and AG drafted the manuscript; MG, JS, VS and CZ extracted and checked the data; UM and AG provided statistical and methodological input; KP provided pathological analysis; ISW and CR contributed to the manuscript and its revision. All authors revised and approved the manuscript.

## Pre-publication history

The pre-publication history for this paper can be accessed here:


